# The Impact of Double-Fortified Salt Delivered Through the Public Distribution System on Iodine Status in Women of Reproductive Age in Rural India

**DOI:** 10.1093/cdn/nzab028

**Published:** 2021-03-30

**Authors:** Ujwala Godbole, Mahesh Basantani, Subhash Yadav, Nachiket Godbole, Sukhanshi Khandpur, Madan Godbole, Sana Raza, Mduduzi N N Mbuya, Lynnette M Neufeld

**Affiliations:** Institute of Bioscience and Technology, Shri Ramswaroop Memorial University, Barabanki, Uttar Pradesh, India; Institute of Bioscience and Technology, Shri Ramswaroop Memorial University, Barabanki, Uttar Pradesh, India; Department of Endocrinology, Molecular Medicine, and Biotechnology, UP-USI Coalition, Sanjay Gandhi Postgraduate Institute campus, Lucknow, India; Department of Endocrinology, Molecular Medicine, and Biotechnology, UP-USI Coalition, Sanjay Gandhi Postgraduate Institute campus, Lucknow, India; Department of Endocrinology, Molecular Medicine, and Biotechnology, UP-USI Coalition, Sanjay Gandhi Postgraduate Institute campus, Lucknow, India; Department of Endocrinology, Molecular Medicine, and Biotechnology, UP-USI Coalition, Sanjay Gandhi Postgraduate Institute campus, Lucknow, India; Department of Endocrinology, Molecular Medicine, and Biotechnology, UP-USI Coalition, Sanjay Gandhi Postgraduate Institute campus, Lucknow, India; Global Alliance for Improved Nutrition, Washington, DC, USA; Global Alliance for Improved Nutrition, Geneva, Switzerland

**Keywords:** double fortified salt, iodized salt, urinary iodine concentration, salt iodine content, women of reproductive age

## Abstract

**Background:**

Double-fortified salt (DFS) with iron and iodine has been demonstrated to be efficacious but questions of unintended effects on the gains in salt iodization remain. The main cross-sectional study based on the use of DFS over 1 y showed a reduction in iron deficiency risk. Whether the programs and the levels of added iron can adversely affect iodine status is yet to be established.

**Objectives:**

We hypothesized that the addition of iron to iodized salt can adversely affect iodine status in women of reproductive age (WRA).

**Methods:**

A cross-sectional substudy was conducted in 4 matched-pair adjacent districts of rural Uttar Pradesh, India, in 2019. Under the public distribution system (PDS), DFS was available for 1 y through Fair Price Shops, in the 2 DFS supply districts (DFS-SDs). In these districts, iodized salt was also available in the market. In the 2 compared DFS nonsupply districts (DFS-NSDs), only iodized salt was available. In the substudy, participants included WRA (*n *= 1624) residing in rural areas of the selected districts. Iodine content in urine and salt samples was measured in each of the groups.

**Results:**

Significantly fewer women from the DFS-SDs had median urinary iodine concentration values indicative of moderate to mild iodine deficiency compared with the women from the DFS-NSDs. The salt purchase pattern and iodine content revealed that significantly fewer (21.99%) households in the DFS-SDs were purchasing inadequately iodized crystal salt, compared with 36.04% households in the DFS-NSDs.

**Conclusions:**

The data reject the working hypothesis and suggest a beneficial effect of the DFS program on the iodine status in WRA, thereby supporting a recommendation of DFS supply through the PDS.

## Introduction

Iodine deficiency disorders (IDDs) and iron deficiency anemia (IDA) coexist in many parts of India (1). This is true for a majority of South Asian Association for Regional Cooperation countries, which include Afghanistan, Bangladesh, Bhutan, India, Maldives, Nepal, Pakistan, and Sri Lanka, where 24.89% of the total world population resides (2). Universal salt iodization (USI) has been implemented in India since 1987, and at the national level (3), the household coverage of salt with iodine content ≥15 ppm was 76.3%. Salt with some amount of iodine ≥5 ppm at the household level was 92.4%. Taken together, these levels indicate a public health success story (4, 5). At the national level, the median urinary iodine concentration (UIC) was 173.4 µg/L for pregnant women, 172.8 µg/L for lactating women, and 178.0 µg/L for nonpregnant nonlactating women. Across place of residence, the median UIC in urban areas was slightly higher (180.2 µg/L) compared with rural areas (168.9 µg/L) (4, 5). This success motivated the introduction of double-fortified salt (DFS) containing both iron and iodine (5–7). Both iodine and iron deficiencies constitute major problems that demand a unified solution at the community and international nutrition levels.

The literature is replete with studies demonstrating the efficacy of introducing DFS, albeit in vulnerable populations, with tangible benefits to ameliorate IDA (8–17), with 1 exception (18). The Food Safety and Standards Authority of India (FSSAI), an autonomous body established under the Ministry of Health & Family Welfare, Government of India, based on expert advice and literature support (15, 16), has reached a consensus on the use of encapsulated ferrous fumarate as a fortificant of choice with permitted additives (19, 20). The range of iron has been prescribed between 850 and 1100 ppm, in a notification issued by the FSSAI (21). Pilot projects have been launched in the states of Rajasthan, Madhya Pradesh, Uttar Pradesh, and Jharkhand. At present, the information that DFS improves circulating ferritin concentrations in women of reproductive age (WRA), needs to be supplanted with evidence that the introduction of DFS does not risk compromising the gains achieved through either USI or the public distribution system (PDS). Its inclusion as an essential commodity under the PDS has been recommended without providing adequate data on iodine status under real-life conditions (22).

Presently, India is at a crossroads of decision-making regarding DFS as an alternate choice to promote iodine and iron nutrition, for several reasons that include: *1*) cost of DFS being unaffordable for the poorer segments of the population; *2*) effectiveness against iron deficiency or anemia, through programs delivered at scale (effectiveness studies), has not yet been proven beyond doubt; and *3*) plausible iodine–iron interaction is not fully established for encapsulated ferrous fumarate used in fortification.

Because the iron to iodine ratio in DFS is set at ∼45:1, left to their own devices, iron and iodine might interact. Encapsulation appears to prevent the interaction between the two, ensuring DFS palatability and the bioavailability of the nutrients (20, 22). It is contemplated that DFS, if well consumed, will be a replacement for iodized salt (21, 23). The physiological interaction between iodine and iron forms the basis for addition of iron to iodized salt in many parts of the world, for improved iodine uptake by the thyroid gland (22). Iron deficiency exacerbates the impairment of thyroid hormone synthesis, storage, and secretion by reducing the activity of heme-dependent thyroid peroxidase (24, 25). However, the effect of daily consumption of iron through DFS on iodine status, especially in WRA, has not been studied. The present study was aimed at investigating the effect of DFS consumption on iodine status in WRA, under real-life conditions.

## Methods

### Study design

This study was focused on WRA residing in 4 adjacent districts of Uttar Pradesh. These districts were not known to be iodine deficient, and >70% of the population can be assumed to have received iodized salt under the USI program, for the past 2 decades. The Uttar Pradesh state government agreed to implement the distribution of DFS containing both iron and iodine, through the PDS, with the primary goal of assessing its impact on moderately prevalent IDA in 10 districts (19). The PDS is a social safety-net program in India, and PDS shops in the state of Uttar Pradesh distribute rations that include subsidized rice, wheat, and kerosene to eligible households every month. PDS eligibility is determined by the state government, and the lowest income households are categorized as Antyodaya Anna Yojana (AAY) cardholders and receive DFS at Indian Rupee (INR) 3/kg (one-sixth of the market price). Slightly better-off households are categorized as Priority Household (PHH) cardholders, and procure and receive DFS at INR 6/kg.

The current cross-sectional study was carried out as a substudy of an evaluation of the effectiveness of DFS, distributed for 12 mo through the PDS, in Uttar Pradesh, India (19). Only 2 districts, namely Etawah and Auraiya, met the evaluability threshold of 50% DFS utilization chosen as an a priori criterion, and were included, along with their 2 matched adjacent boundary comparison districts of Mainpuri and Kannauj (19). In all these districts, iodized salt was freely available in the market place. In a previous study, the prevalence of adequate iodine salt in the study population was observed to be 76.3% (4, 5), assuming 80% sensitivity and 80% specificity, to detect adequate iodine salt in the study samples where the null hypothesis was assumed to be 75%. At minimum 2-sided 95% CI and 80% power of the study, the minimum required sample size was 750. After incorporating the design effect of 2, the revised target sample size was 1500. The sample size was estimated by using PASS-16 software (NCSS Statistical Software). The sample size from DFS supply districts (DFS-SDs) was kept at a 2:1 ratio because of the high coverage but low usage of the DFS program observed after the implementation of DFS distribution in DFS-SDs (25). Four teams visited the villages daily for the main survey covering WRA. To assess whether the higher UIC seen in WRA from DFS-SDs was due to DFS or other extraneous factors, we investigated the types of salt being used, and observed the consumption of either powdered or crystal salt (big crystal). The team members visiting village households were instructed to request salt samples from every third household after explaining the purpose of such collection.

Inclusion criteria included: *1*) nonpregnant women aged 18–49 y (WRA); *2*) households in the 2 high-performing districts and their adjacent DFS nonsupply districts (DFS-NSDs); and *3*) having a ration card for PDS. Exclusion criteria were: individuals with physical and/or mental impairments that impeded measurements or provision of informed consent. The impact assessment of iodine consumption and its status in the WRA sampling frame indicated that the collection of salt and urine samples from every third household would satisfy the 2-group community-based study design. In the original study of impact of DFS on iron deficiency and anemia in WRA in rural households, the villages were randomly selected and divided into 4 segments (23). Then from each segment, every fourth household was selected using systematic sampling. Among those selected households, we selected every third household for inclusion in our substudy.

The study was authorized by the State Ministry of Health authorities, and the data collection and analysis protocols were reviewed and approved by the Institutional Review Board of Sanjay Gandhi Postgraduate Institute, Lucknow, India. The evaluation is registered with 3ie's Registry for International Development Impact Evaluations (RIDIESTUDY-ID-58f6eeb45c050). Data were collected between May and July, 2019.

### Laboratory methods

#### Qualitative detection of iodine and iron in DFS

Qualitative detection of iron and iodine in DFS was done using thiocyanate and acidic starch reagents, respectively. Appearance of blue and brick-red color was indicative of the presence of iodine or iron, respectively. Findings were recorded as: *1*) Iron-Yes, Iodine-Yes, and *2*) Iron-No, Iodine-Yes. The samples that tested positive for both iodine and iron (DFS) as well as those found positive for iodine only (iodized salt), were subjected to titration for the quantitative estimation of iodine content.

#### Salt iodine estimation in iodized salt and DFS

Following the conventional iodometric titration used for the estimation of iodine in iodized salt, 10 g iodized salt was dissolved in 50 mL distilled water, and 1 mL 2N sulfuric acid (H_2_SO_4_) was added, followed by the addition of 5 mL 10% potassium iodide (KI). The reaction mixture was kept in the dark for 10 min, and the iodine liberated was estimated by titration with 0.005 M sodium thiosulfate (Na_2_S_2_O_3_), using a starch indicator towards the end point of titration (26). In case of samples confirmed as DFS by use of thiocyanate and acidic starch reagents, use of H_2_SO_4_ was replaced with phosphoric acid (H_3_PO_4_) (26, 27).

#### Urinary iodine concentration estimation

Interfering substances in urine samples were removed by ammonium persulfate digestion. In microplates, urinary iodide was measured by the Sandell–Kolthoff method of conversion of Ce^4+^ (yellow) to Ce^3+^ (colorless) by As^3+^ in acid medium (28–30). The reaction was read by a microplate spectrophotometer, at 405 nm (31, 32).

### Statistical analysis

Because UIC data do not follow a normal distribution, we used a nonparametric alternative to a 2-sample *t* test, the Mann–Whitney *U* test, to study the statistical significance of differences in urinary iodine between the 2 groups. Values of urinary iodine were reported as median and 95% bootstrap CI. Upper confidence limits and lower confidence limits were calculated based on the SE. A 2-sample test for proportions was used for categorical variables. Our primary aim was to explore the unadjusted treatment level differences, but we additionally adjusted the analysis for pairing of districts using generalized linear models (GLMs) to confirm the robustness of our findings. All analyses were performed using Stata14.1 (StataCorp).

## Results

Owing to the availability of >1 WRA in a household, the 1624 sample size obtained in this study was a little higher than estimated. The sociodemographic characteristics of the samples are presented in **Table 1**, stratified by the DFS supply. Most of the respondents were Hindu, food secure, and had at least primary education. The DFS-SDs and DFS-NSDs were comparable across all characteristics, except the Fair Price Shop card type. PDS eligibility is determined by the state government, and the lowest-income households are categorized as AAY cardholders, whereas slightly better-off households are categorized as PHH cardholders. However, this slight imbalance did not reflect in the relative wealth, which was balanced. **Table 2** depicts the number of urine and salt samples collected and protocol adherence, as illustrated by the ratio of 2:1 among DFS-SDs and DFS-NSDs, per design. The minor discrepancies can be explained by households with >1 WRA who provided urine samples, and by households with insufficient salt samples to provide to the visiting team members. Such discrepancies were found in <4% of the total collection of samples.

**TABLE 1:** Sociodemographic characteristics of the households and individuals sampled in the DFS supplied and DFS nonsupplied districts^1^

Characteristic	DFS supplied (*n *= 1153)	DFS nonsupplied (*n *= 499)	*P* value^2^
FPS card type, *n* (%)			
Antyodaya Anna Yojana (AAY)	238 (20.6)	85 (17.0)	<0.01
Priority Household (PHH)	915 (79.4)	414 (83.0)	
Purchase provisions from FPS, *n* (%)			
1	1126 (97.7)	482 (96.6)	0.69
2	27 (2.3)	17 (03.4)	
Education level of respondents, *n* (%)			
No education	323 (28.0)	153 (30.7)	0.11
Primary school	154 (13.4)	67 (13.4)	
Middle or high school	433 (37.6)	155 (31.1)	
Higher	220 (19.1)	112 (22.4)	
Missing	23 (2.0)	12 (2.4)	
Education level of household head, *n* (%)			
No education	245 (21.3)	124 (24.9)	0.28
Primary school	121 (10.5)	61 (12.2)	
Middle or high school	563 (48.8)	222 (44.5)	
Higher	224 (19.4)	92 (18.4)	
Religion of household head			
Hinduism	1106 (95.9)	466 (93.4)	0.27
Muslim	47 (4.1)	33 (6.6)	
Number of people in household, mean ± SE	5.95 ± 0.1	6.29 ± 0.1	0.02
Wealth quintile, *n* (%)			
Lower	230 (20.0)	95 (19.0)	0.17
Lower middle	219 (19.0)	99 (19.8)	
Middle	251 (21.8)	83 (16.6)	
Upper middle	215 (18.7)	111 (22.2)	
Upper	238 (20.6)	111 (22.2)	
Household hunger score, *n* (%)			
Food secure	827 (71.7)	359 (71.9)	0.50
Moderate food insecurity	260 (22.6)	121 (24.3)	
Severe food insecurity	66 (5.7)	19 (3.8)	

1 Values are mean ± SE or *n* (%). DFS, double-fortified salt; FPS, Fair Price Shop.

2 *P* values indicate difference between DFS supplied and DFS nonsupplied districts, and incorporate design effect.

**TABLE 2:** Details of numbers of urine and salt samples collected from households^1^

Category	District name	Urine^2^	Salt^2^
A: DFS-SD	Etawah	554	512
	Auraiya	615	610
	Total	1169	1122
B: DFS-NSD	Kannauj	225	247
	Mainpuri	260	255
	Total	485	502
A/B ratio^3^		2.41:1	2.23:1
	Grand total	1654	1624

^1^DFS-NSD, double-fortified salt nonsupplied district; DFS-SD, double-fortified salt supplied district; WRA, women of reproductive age.

2 Mismatch between urine and salt samples: a larger number of urine samples from households indicates >1 WRA in household and their insistence for testing; a few households refused to provide salt samples.

^3^DFS-SD/DFS-NSD ratio (A/B) was greater than the proposed ratio of 2:1.

UIC values in women from DFS-NSD (median UI value: 89.75 µg/L; 95% CI: 81.76, 97.73 µg/L) were significantly reduced by two-fifths from the UIC values in women overall in DFS-SDs (median UI value: 224.25 µg/L; 95% CI: 210.26, 238.23 µg/L) (*P *< 0.01).

**FIGURE 1:**
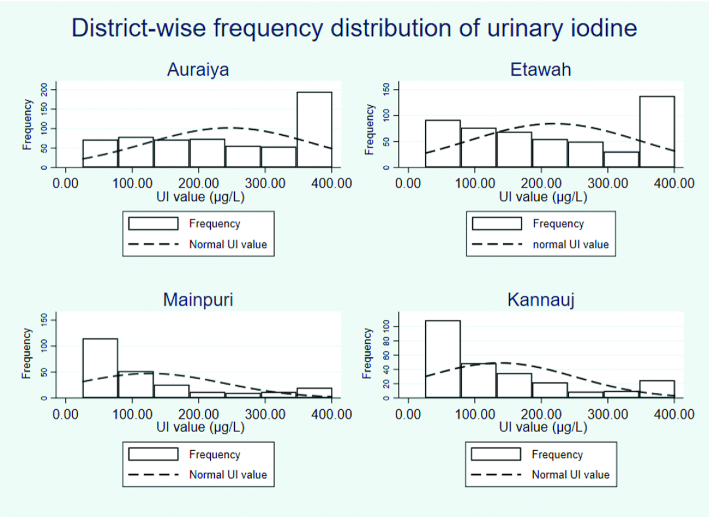
District-wise frequency distribution of urinary iodine. Overall median urinary iodine concentrations <100 μg/L were found in 31% of the households under study. Median urinary iodine concentrations <100 μg/L were found in 17% and 23%, respectively, in the households of DFS-SDs of Auraiya and Etawah. However, overall median urinary iodine concentrations <100 μg/L were found in 60% and 51%, respectively, of the households of DFS-NSDs of Mainpuri and Kannauj. DFS-NSD, double-fortified salt nonsupplied district; DFS-SD, double-fortified salt supplied district; UI, urinary iodine.

**FIGURE 2:**
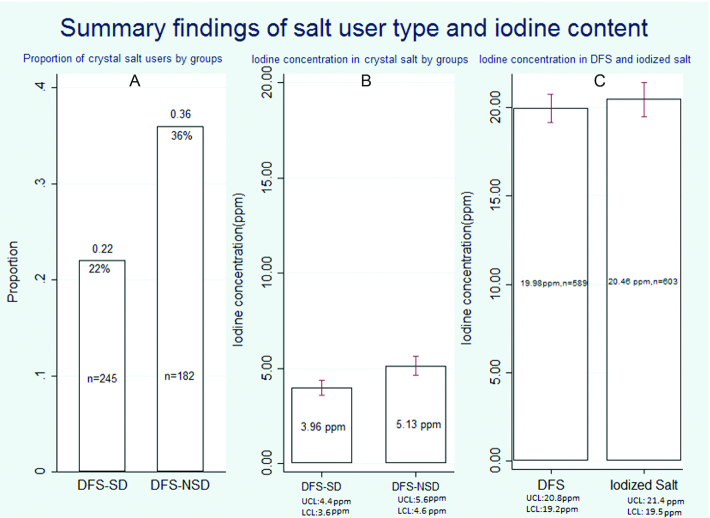
Summary findings of salt user type and iodine content. (A) There was a lower percentage of crystal salt users in DFS-SDs compared with DFS-NSDs (*P *< 0.01). (B) The iodine content of crystal salt indicated inadequate iodination levels whether collected from DFS-SDs or DFS-NSDs although iodine concentration was statistically significantly higher in crystal salt used in DFS-NSDs. (C) The iodine content of powdered salt indicated adequate iodination level in either DFS collected from DFS-SDs or iodized salt from DFS-NSDs. DFS-NSD, double-fortified salt nonsupplied district; DFS-SD, double-fortified salt supplied district; LCL, lower confidence limit; UCL, upper confidence limit.

UIC values were significantly higher in women in the DFS-SDs (median UI value: 226.20 µg/L; 95% CI: 202.30, 250.09 µg/L) than the UIC values in women in districts supplied with crystal salt (median UI value: 145.70 µg/L; 95% CI: 129.08, 162.31 µg/L) (*P *< 0.01) and iodized salt (median UI value: 139.60 µg/L; 95% CI: 121.69, 157.50 µg/L) (*P *< 0.01). It was also observed that 25% of women from DFS-NSDs had UIC values ≤50 µg/L (**Figure 1**). To address whether the higher UIC seen in WRA from DFS-SDs was due to DFS or other extraneous factors, the types of salt being consumed were investigated. The salt types used in the households of study subjects and their iodine content are given in **Figure 2**. This shows that the percentage of crystal salt users is significantly lower in DFS-SDs compared with DFS-NSD households (*P *< 0.01, Figure 2A). The iodine content of powdered salt (DFS or otherwise) from both DFS-SDs and DFS-NSDs indicated an adequate and equal degree of iodination (Figure 2B). In contrast, an inadequate but equal degree of iodination was observed in big crystal salt samples from both DFS-SDs and DFS-NSDs (Figure 2C). Additionally, we explored unadjusted and adjusted [for respondents’ age, respondents’ education, household head education, respondents’ religion and housing, wealth quintile, hunger score, and WASH (water, sanitation, and hygiene) index quintile] GLM regression analyses. Comparing WRA in DFS-SDs with DFS-NSDs, we found that UIC values were 93.5 μg/L (95% CI: 71.3, 115.8 μg/L; *P *< 0.0001) higher in unadjusted, and 93.3 μg/L (95% CI: 72.2, 114.4 μg/L; *P *< 0.0001) higher in adjusted analyses.

## Discussion

We assessed the average effect of the DFS program on the iodine status of WRA in rural India. The study results prima facie indicate significant improvement in iodine status of WRA from DFS-SDs. The sample size obtained in the study was a little higher than expected, the DFS-SD/DFS-NSD ratio (A/B) was greater than the proposed ratio of 2:1. The larger number of urine samples from the households indicated >1 WRA in the household and their insistence for testing; however, a few households refused to give salt samples. The sample collection represents a real-life situation. We observed a lower prevalence of moderate to mild iodine deficiency in WRA in DFS-SDs compared with DFS-NSDs, as measured through median UIC. It is difficult to explain the 2.5-fold higher UIC concentration in DFS-SD group WRA. These findings are likely, at least in part, explained by the replacement of crystal (un-iodized or inadequately iodized salt) for adequately iodized DFS. The median UIC from our study in DFS-SDs, of 224.25 µg/L for WRA, indicates iodine sufficiency and is concurrent with the reported median UIC (167.1 µg/L) for the state of Uttar Pradesh (2). A median UIC ≤100 µg/L in spot urine samples was indicative of deficiency in the studied population (29, 30). Additionally, no more than 20% of the samples should have a UIC <50 µg/L. The range in which the median falls is more important than the precise number (30–32). Interestingly, despite the lower use of crystal salt in the DFS-SDs than the DFS-NSDs, its use in the DFS-SDs was still much higher than the Indian average (3, 4). DFS consumption is unlikely to compromise iodine status, and on the contrary, can contribute to improving it if the trend toward replacement of poorly iodized crystal salt is confirmed. It is important to clarify that these are average effects, but confirmed through GLM regression; however, further adjustments using econometric methods will be needed. It is also unlikely that the observed differences are related to differences at baseline: in the full baseline survey, iodine deficiency in the treatment districts (albeit including those that did not meet the evaluability criteria) was 51%, compared with 57% in comparison districts (*P *< 0.07) (25). Although not related to the primary aim of the study, that is, the assessment of DFS distribution impact on iodine status, the observation that a substantial proportion of households still make use of poorly iodinated crystal salt is a worrisome finding. This warrants the monitoring of salt iodine concentrations under the USI program and also calls for monitoring of either UIC or total goiter rate in school children, on a regular basis.

Seeking reasons why a larger number of households were purchasing poorly iodinated crystal salt, it became clear that the salt supply in these districts came from the adjoining salt-producing state of Rajasthan via road transport, rather than via rail from the far-off state of Gujarat, with the former being less amenable to monitoring but more readily available to consumers at a cheap price (33). The primary point is that subsidized DFS obtained through the social protection system of the PDS program could mitigate this well-documented risk to USI programs. The results fulfill 1 of the criteria laid down in the FDA's 1980 policy guidelines, which suggests that fortificants should not cause any unintended consequences (34). The strength of this study lies in the fact that both salt and urine samples were collected under real-life situations, which avoids the bias that can affect school surveys. Based on our results, we conclude that DFS has a salutary effect on the iodine status of WRA, and recommend its continued supply and use because it is enhancing the achievements of USI.

## Notes

This study was funded by the Bill & Melinda Gates Foundation. The funders approved the study design but had no role in the data collection, data analysis, data interpretation, or writing of the report.

Author disclosures: The authors report no conflicts of interest.

Data described in the manuscript will be made available upon formal request.

Abbreviations used: AAY, Antyodaya Anna Yojana; DFS, double-fortified salt; FSSAI, Food Safety and Standards Authority of India; GLM, generalized linear model; IDA, iron deficiency anemia; IDD, iodine deficiency disorder; INR, Indian rupees; NSD, nonsupply district; PDS, public distribution system; PHH, Priority Household; SD, supply district; UIC, urinary iodine concentration; USI, universal salt iodization; WRA, women of reproductive age.
